# Genetic algorithm with a crossover elitist preservation mechanism for protein–ligand docking

**DOI:** 10.1186/s13568-017-0476-0

**Published:** 2017-09-13

**Authors:** Boxin Guan, Changsheng Zhang, Jiaxu Ning

**Affiliations:** Key Laboratory of Medical Image Computing of Ministry of Education, Northeastern University, Shenyang, 110819 People’s Republic of China

**Keywords:** Protein–ligand docking, Pharmaceutical development, Genetic algorithm, AutoDock, Crossover elitist preservation

## Abstract

Protein–ligand docking plays an important role in computer-aided pharmaceutical development. Protein–ligand docking can be defined as a search algorithm with a scoring function, whose aim is to determine the conformation of the ligand and the receptor with the lowest energy. Hence, to improve an efficient algorithm has become a very significant challenge. In this paper, a novel search algorithm based on crossover elitist preservation mechanism (CEP) for solving protein–ligand docking problems is proposed. The proposed algorithm, namely genetic algorithm with crossover elitist preservation (CEPGA), employ the CEP to keep the elite individuals of the last generation and make the crossover more efficient and robust. The performance of CEPGA is tested on sixteen molecular docking complexes from RCSB protein data bank. In comparison with GA, LGA and SODOCK in the aspects of lowest energy and highest accuracy, the results of which indicate that the CEPGA is a reliable and successful method for protein–ligand docking problems.

## Introduction

Protein–ligand docking is one of the most important methods in structure-based pharmaceutical development (Brooijmans and Kuntz 2003; Huang and Zou 2010; Jug et al. 2015; Moitessier et al. 2008; Zhao et al. 2014, 2016), and it is also an important approach for large-scale virtual screening. With the development of X-ray technology, the three-dimensional structure of docked conformations has been obtained so that protein–ligand docking has more practical significance. Through the establishment of protein–ligand docking model, and researching the interaction the receptor and the ligand, to discover and design a more effective, more ideal drugs. The process of molecular docking is to search conformations of the proteins and the ligands with lowest energy. The ligands are placed at the active site of the protein receptors, and reasonable orientations and conformations are sought to match the shape and interaction of ligands and receptors. The active binding site refers to a specific small region in the receptors, which is composed of a small number of amino acid residues on the side chain. The optimized target energy value of molecular docking is obtained by calculating the interaction between the ligands and the binding region of the receptors.

Scoring function (Hu et al. 2004; Huey et al. 2006; Jain 2006; Muryshev et al. 2003) and search algorithm (Blum et al. 2011; Lόpez-Camacho et al. 2015) are two important parts in the process of protein–ligand docking. The scoring function which is a force field to evaluate the energy of the docking conformation is helpful to explore the binding model receptors and ligands. Reasonable scoring function not only can correctly assess the docking results, but it also can distinguish the difference between the results of different docking (Bharatham et al. 2014; Li et al. 2015).

The search algorithm is to find out the optimal binding mode between small ligand and its receptor protein around binding site. Some algorithms have been shown to be very effective for solving the protein–ligand docking problem, and some researchers have improved the power of these docking methods. For example, simulated annealing (SA) (Goodsell and Olson 1990), Genetic algorithm (GA) (Cao and Li 2004; Jones et al. 1997; Thomsen 2003), Lamarckian genetic algorithm (LGA) (Fuhrmann et al. 2010), SODOCK (Chen et al. 2007; Jason et al. 2008; Ng et al. 2015), and artificial bee colony algorithm (ABC) (Uehara et al. 2015). However, to develop an efficient and reliable search algorithm is still a challenge for docking problem.

The parents of the elitist individual in original genetic algorithm are not retained, which lead to good genes of the parents do not continue produce to excellent individual through crossover operation. In the article, a new evolutionary algorithm, namely genetic algorithm with crossover elitist preservation (CEPGA), is presented to overcome the shortcoming. The introduction of the crossover elitist preservation (CEP) mechanism can improve the speed of operation and ensure that the optimal solution is not abandoned. The next generation is better for the competition of the elitist parents and their offspring. Moreover, a local search which can select a optimal solution in the near space of the current solution is incorporated into the GA.

AutoDock is a protein–ligand docking software developed by Morris et al. of Scripps Research Institute in the United States. AutoDock (Kitchen et al. 2004; Morris et al. 2009) is a free and open source docking software, and it is also the most widely used automated docking program. The software first produces the grid of the binding site, and then uses the search algorithm to find the best combination of the receptor and the ligand, and finally evaluates the conformation by means of the scoring function. AutoDock 4.2.6 is used as an experimental environment in this paper. The semi-empirical free energy force field that is based on a overall thermodynamic model which can convert intramolecular energy into binding and predictive free energy in AutoDock 4.2.6 is used as a scoring function in the experiments of the paper. To study the capability of the presented method, genetic algorithm crossover elitist preservation mechanism (CEPGA), it has been tested on a set of different protein–ligand complexes from RCSB protein data bank (PDB) (http://www.rcsb.org/pdb) (Berman et al. 2002) and compared to GA, LGA, SODOCK, and ABC.

## Materials and methods

### Standard genetic algorithm

Genetic algorithm (GA) is a method by simulating Darwin’s theory of natural evolution to search for the optimal solution. Genetic algorithm starts from a population contains potential solutions of a specific problem. Each encoding corresponds to a solution for the problem, and it called a individual or chromosome. Then with the help of selection, crossover, and mutation produces a new population. This process results in that the population evolves from generation to generation to get more and better approximate solutions according to the principle of survival of the fittest. The best individual which is decoded in the last population can be used as an optimal solution. On the basis of the ability of the individual to adapt to the environment, selection decides the survival or the elimination of the individual. The selection operation enables the individuals with higher fitness which is evaluated using the scoring function to be preserved with greater probability, so that the population converges to the global optimum at the fastest speed. Sort selection is a ranking of all individuals according to their fitness values and determines the probability of individuals being selected, it is used in GA for protein–ligand docking. The process in which individuals randomly pair up, exchange part of their chromosomes at a probability, and form new individuals is called crossover. One point crossover, an intersection is randomly selected and two individuals swap at the front or back of the point to produce a new individual, is adopted as crossover operator of GA for protein–ligand docking. The so-called mutation, which is a number of accidental factors, causes the genes in individuals are randomly transformed at a certain probability and produces new individuals. For the protein–ligand docking problem, GA is real code, so real mutation is used as mutation operator.

### Genetic algorithm with crossover elitist preservation mechanism

The crossover of genetic algorithm, first of all, two relative paired individuals are determined based on specific principles. Then, they exchange some genes in a specific way to form two new individuals. The purpose of crossover is to keep the good genes of the parent generation and generate a lot of new individuals. However, the pairing of the individuals is random in the parent generation, and the randomness plays an ineffective role in the global search. The excellent individuals of the previous generation have not been retained due to the randomness, and the individuals may not be as good as the previous generation. Accordingly, a novel crossover strategy is introduced.

In the method, X_0_, X_father_ and X_mother_ are introduced. X_0_ represents elitist individual, X_father_ represents the father of elitist individual, and X_mother_ represents the mother of elitist individual. When the current solution is better than any other solutions before, the current solution is defined as X_0_ and X_father_ and X_mother_ of X_0_ are preserved. The saved value of the parents are used for the next crossover operation. With the development of the algorithm, using good values of X_father_ and X_mother_ instead of other values for crossover, the search algorithm are gradually efficient. The new method is called crossover elitist preservation mechanism and abbreviated as CEP.

**Fig. 1:**
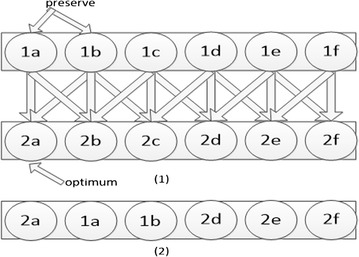
Diagram of CEP. (1) The individuals of the previous generation pair and cross to generate the individuals of the next generation. The optimum individual 2a serves as an elitist individual, and its parents are preserved. (2) The adjusted individuals of the next generation after CEP

Example: suppose CEPGA randomly generates six individuals, 1a, 1b, 1c, 1d, 1e and 1f, respectively, in the first generation. In Fig. 1 (1), six new individuals are produced by crossover operator of GA in the second generation, such as 1a and 1b cross to generate 2a. If the individual 2a is the current optimal solution, the parents of the elitist individual, 1a and 1b, are preserved. Because the genes of the parents of the elitist individual are excellent, they may be more likely to reproduce elitist individuals. Then the preserved individuals, 1a and 1b, replace the individuals, 2a, 2b, 2c, 2d, 2e, and 2f, in the second generation. 2a as the elitist individual can not be replaced. Two random individuals of the remaining five individuals are selected in the second generation, such as 2b and 2c, and then replace them with 1a and 1b. In Fig. 1 (2), 2b and 2c are replaced by saved individuals 1a and 1b, so the next generation is 2a, 1a, 1b, 2d, 2e, 2f.

By using the CEP, the parents of the elitist individual and the population of current generation are combined to make the gene quality of the population better, ensures that the genes of good individuals are not discarded during evolution, and maintain that the genes of the best individuals in the population can pass on to the next generation. For protein–ligand docking, the number of elitist individuals is θ*the number of population, where θ is a particular adjustable number (the range is 0.01–0.1). Hence, the number of the parents of elitist individuals is 2θ*the number of population. The parents of the elitist individuals are preserved, and they replace individuals of current generation except the elitist individuals.

Local search is an algorithm chooses an optimal solution in the near solution space of the current solution, until it reaches a local optimal solution. The basic idea of local search algorithm: search direction is carried out along the direction of the solution of the target. If a solution is not a local optimum, the local search can get a optimal solution in its near space. In the search process, the locally strong search algorithm always selects the neighborhood of the current solutions. The local search is also added to the novel algorithm (CEPGA) in order to improve the efficiency.

**Fig. 2:**
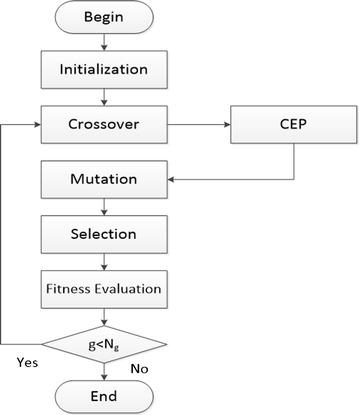
Block diagram of CEPGA. The basic process of CEPGA is showed in the figure. CEP was applied after crossover so that the genes of the new population are excellent

The pseudo-code and the block diagram of CEPGA is showed in Table 1 and Fig. 2, respectively. CEPGA begins with a random initialized population. Then, the next population is reproduced after crossover, CEP (steps 04–11), mutation and selection. From the second generation, elitist individuals with good genes are reproduced, and the parents of these elitist individuals are preserved. The preserved individuals of parent generation and the individuals of sub-generation are combined to form a new parent population. The introduction of the crossover elitist preservation strategy can increase the sampling space and the competition among individuals. It is easier to get a better solution through the competition among the elitist individuals in the new formed generation. This process continues until a specific termination condition is reached. The above steps ensure that the best genes are not destroyed and the algorithm evolves toward the direction of the optimal solution.

**Table 1:**
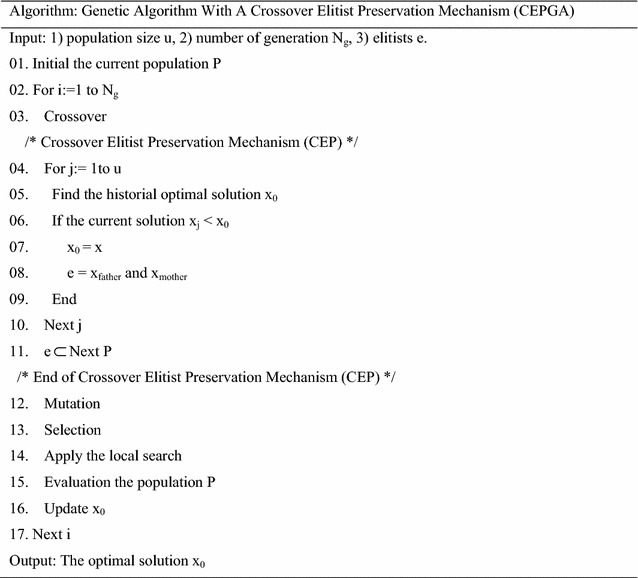
Pseudo-code of CEPGA

## Results

To value the impact of the presented algorithm, the performance found by CEPGA with GA, LGA, SODOCK and ABC is compared. The semi-empirical free energy force field described above is used in all experiments in this paper. In order to maintain the diversity of the protein–ligand X-ray structures, theses instances should have a wide span of the number of rotatable bonds in ligands. Sixteen protein–ligand X-ray structures (Hu et al. 2004) with 0–15 rotatable bonds in ligands are chosen from RCSB protein data bank (Berman et al. 2002) (http://www.rcsb.org/pdb) to compare the capability of the docking techniques.

### (1) 3ptb beta-trypsin/ben (benzamidine)

Beta-trypsin is a kind of protease, which is extracted from the pancreas of cattle, sheep and pigs. Benzamidine is an inhibitor, and it is often used to prevent proteolytic degradation of proteins.

### (2) 1aha alpha-momorcharin/ade (adenine)

Alpha-momorcharin is extracted from the seeds of *Momordica charantia*. Adenine is a substance in the body.

### (3) 3hvt HIV-1 reverse transcriptase/nvp

HIV-1 reverse transcriptase is the three phosphate enzyme that synthesizes complementary DNA. Nvp is a potent, non-nucleoside reverse transcriptase inhibitor.

### (4) 1phg cytochrome P450-cam/hem (protoporphyrin IX)

Cytochrome P450-cam is a superfamily of heme-thiolate proteins, it is involved in the metabolism of endogenous and exogenous substances. Protoporphyrin IX is purple brown crystalline powder, soluble in methanol, insoluble in water, chloroform, ether and acetone.

### (5) 2mcp McPC-603/pc (phosphocholine)

McPC-603 is a phosphocholine-binding mouse myeloma protein. Phosphocholine is an intermediate in the synthesis of phosphatidylcholine in tissues.

### (6) 1stp streptavidin/btn (biotin)

Biotin, also known as vitamin H or coenzyme R, is a water-soluble B-vitamin. Streptavidin/Biotin is one of the most tightly binding noncovalent complexes. 窗体顶端

Streptavidin is a kind of protein that gained from streptomyces, and it has a similar biological characteristic with affinity. Biotin is one of the B vitamins, and it is essential for the normal metabolism of fats and proteins.

### (7) 6rnt ribonuclease T1/ca (calcium ion)

Ribonuclease T1 is a endonuclease that removes the non hybridized RNA region in the DNA–RNA hybrid. Calcium ion is an indispensable ion in the physiological activities of the body.

### (8) 4dfr dihydrofolate reductase/mtx (methotrexate)

窗体顶端

Dihydrofolate reductase is an enzyme that has been used as a drug-target in the building of anti-cancer and other processes. Methotrexate is an substance that has a strong immunosuppressive effect, it can prevent division and proliferation of immune cells.

### (9) 1ett thrombin/4qq

Thrombin is a white to gray amorphous material, and it is generally freeze-dried powder. 4qq is a non-polymer inhibitor.

### (10) 1hri human rhinovirus/s57

Human rhinovirus is a kind of rhinovirus and the main cause of the common cold in humans. S57 is a kind of imidazole.

### (11) 1hvr protease/xk2

Protease is an enzyme that catalyzes protein catabolism, and it can be find in plants, animals, and so on. Xk2 is an small molecule inhibitor that can block or reduce the rate of chemical reaction.

### (12) 4hmg hemagglutinin/sia (sialic acid)

Hemagglutinin is a substance that results in red blood cells to coagulate. Sialic acids are acidic monosaccharides which are produced at terminal sugars chains.

### (13) 1cdg cyclodextrin glycosyl transferase/mol (maltose)

窗体顶端

Cyclodextrin glycosyltransferase is a bacterial enzyme which has the ability to generate cyclodextrins. Maltose is a substance formed from malt and starch, and it is used as a nutrient and a culture medium.

### (14) 1htf HIV-1 protease/g26

HIV-1 protease is an enzyme that separates newly synthesized polyproteins to their component peptides. G26 is a non-polymer inhibitor. G26 is a kind of amide which is a highly reactive and easily oxidizable perssad.

### (15) 1glq glutathione S-transferase/gtb (S-(P-nitrobenzyl)glutathione)

Glutathione S-transferase is a group of enzymes related to the detoxification function of the liver. S-(P-nitrobenzyl)Glutathione is an important synthesis of glutathione precursor.

### (16) 1tmn thermolysin/nas (2-naphthalenesulfonic acid)

Thermolysin is a biological substance, and it is characterized by the hydrolysis of hydrophobic amino acids at a faster rate. 2-naphthalenesulfonic acid is white crystal or powder, soluble in water, insoluble in alcohol, and it can be used in organic synthesis.

The AutoDock’s PDBQTs of the protein and the ligand are prepared firstly. The PDBQT of the protein is obtained using the following steps: (1) read protein. (2) remove water molecules. (3) add hydrogen. The ligand follow the lowing procedure to get the PDBQT format: (1) read ligand. (2) detect root. (3) choose torsion. (4) set number of torsion.

It is necessary to make sure that the parameters of different search algorithms are equally set up. Therefore, in the three GAs, the population is 50, the number of generations is 27,000, and the energy evaluations is 1.5 × 10^6^ in a docking. In this way, the dockings are terminated by reaching the maximum number of generations. In the SODOCK, the number of particles and immediate neighbors is 50 and 5, respectively; while the maximal number of function evaluations is 1.5 × 10^6^. And in the ABC, the number of the population is 50, and the maximum number of cycles is 1.5 × 10^6^.

Each method is run ten times independent for each protein–ligand docking problem. Table 2 lists the protein–ligand complex names (PDB), the ligand names, the number ligand torsions, the lowest energies and the smallest RMSDs for all 16 test proteins. RMSD is the root mean square deviation between the docking results and the crystal complex, and it is the most important index to evaluate the docking accuracy. It is acceptable if the RMSD is less than 2.0 Å, otherwise the docking is invalid. Through the results table, It is concluded that the CEPGA finds 13 lowest energy of thirteen in the 16 molecular docking complexes. The smallest RMSD found by each of the five search algorithms is 9, 2, 2, 1, and 2 using CEPGA, LGA, GA, SODOCK, and ABC respectively.

**Table 2:** Lowest energy and smallest RMSD results of five compared algorithms

		CEPGA		LGA		GA		SODOCK		ABC	
PDB	Ligand (torsions)	Energy	RMSD	Energy	RMSD	Energy	RMSD	Energy	RMSD	Energy	RMSD
3ptb	ben (0)	−11.72	1.90	−11.46	1.92	−10.31	1.66	−11.57	2.00	−10.90	1.97
1aha	ade (1)	−15.32	0.89	−16.10	0.45	−15.16	1.28	−14.95	1.44	−13.90	1.80
3hvt	nvp (2)	−17.90	0.30	−17.22	0.33	−15.73	0.43	−16.78	0.58	−15.60	0.55
1phg	hem (3)	−9.32	0.64	−8.56	0.80	−7.46	1.20	−8.95	1.54	−7.95	1.67
2mcp	pc (4)	−9.10	1.20	−8.22	1.33	−7.76	1.46	−7.72	1.42	−7.80	1.54
1stp	btn (5)	−13.57	0.90	−13.37	1.65	−11.03	1.84	−13.52	1.00	−13.17	1.68
6rnt	ca (6)	−9.32	0.58	−9.13	0.70	−8.58	0.69	−9.12	1.95	−8.90	1.55
4dfr	mtx (7)	−12.12	1.90	−11.44	1.23	−10.01	0.95	−11.34	1.60	−10.21	1.97
1ett	4qq (8)	−14.21	1.29	−13.89	1.38	−11.42	1.62	−12.06	1.56	−12.70	1.70
1hri	s57 (9)	−10.89	1.38	−10.21	1.87	−9.67	1.80	−10.31	1.68	−10.13	1.67
1hvr	xk2 (10)	−31.06	0.64	−30.85	0.62	−21.95	1.68	−29.29	0.68	−28.64	0.85
4hmg	sia (11)	−10.32	1.89	−10.09	1.70	−8.44	1.69	−10.08	1.36	−9.80	1.54
1cdg	mol (12)	−8.70	1.45	−8.22	1.94	−7.32	1.69	−8.45	1.80	−7.13	1.12
1htf	g26 (13)	−21.48	1.27	−20.69	1.33	−18.86	1.46	−21.79	1.42	−19.17	1.96
1glq	gtb (14)	−9.46	1.38	−9.27	1.87	−7.97	1.87	−8.83	1.90	−9.13	1.60
1tmn	nas (15)	−10.29	0.85	−10.11	1.20	−9.68	1.11	−10.62	1.95	−9.37	0.60

**Fig. 3:**
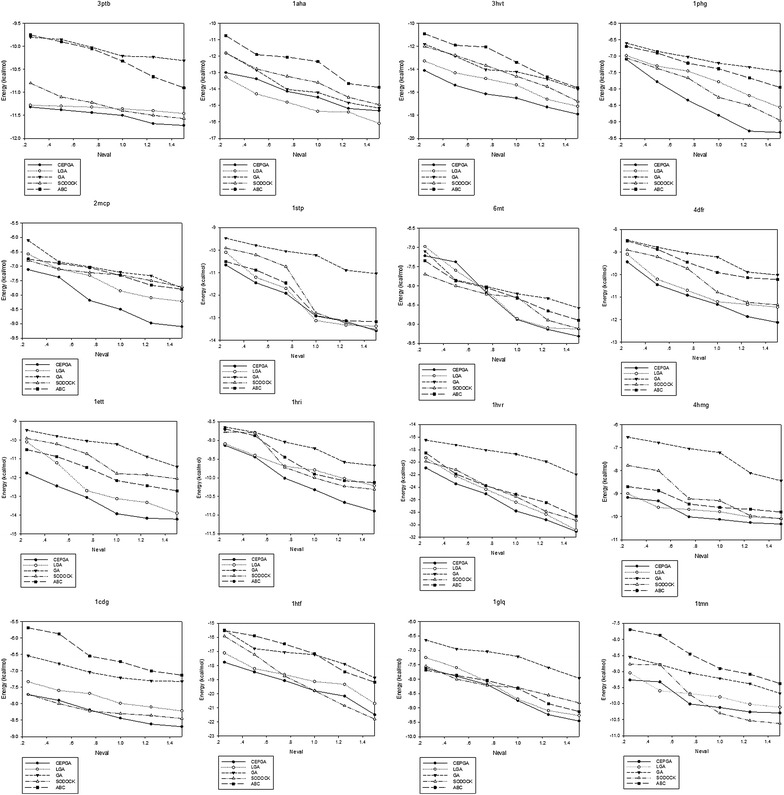
Convergence diagrams of five algorithms in different PDB. The energy is used as the vertical axis and the number of energy evaluations Neval is used as the horizontal axis. The energy values of different Neval are recorded

**Fig. 4:**
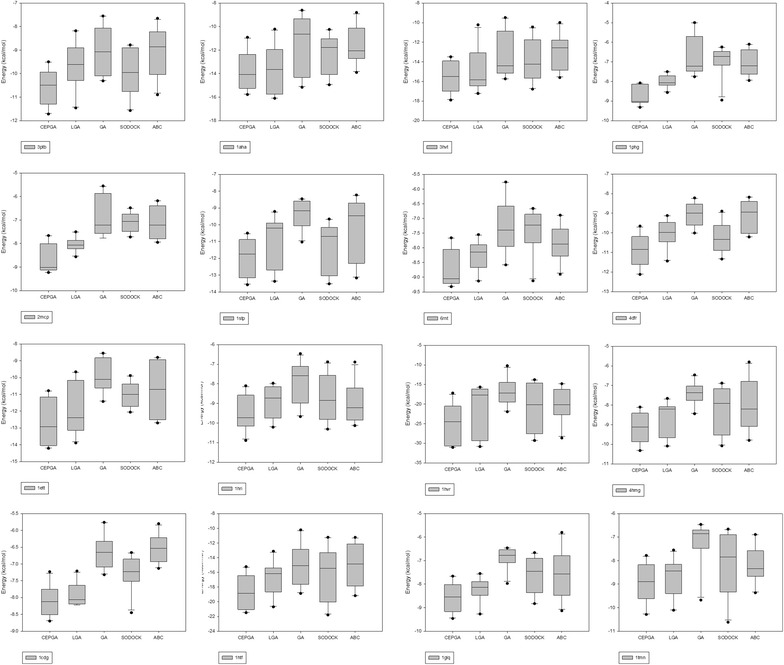
Box plots of five algorithms in different PDB. The energy is used as the vertical axis and the five compared algorithms are used as the horizontal axis. The energy values of the upper edge, the upper quartile, the median, median, the lower four quantile, and the lower edge are recorded, respectively

The convergence diagrams are illustrated in Fig. 3. The experiment records the optimal energy as the vertical axis and the number of energy evaluations when the optimal energy value is evaluated as the horizontal axis. The convergence curve and the convergence period of the algorithm are observed, which provides a reference for the performance evaluation. Figure 4 shows box plots between five compared algorithms in different PDB. The energy values of each PDB are arranged from large to small, and the upper edge, the upper quartile, median, the median, the lower four quantile, and the lower edge are calculated, respectively. Under the confidence level of 0.05, we adopt hypothesis test (Knowles et al. 2006) to demonstrate whether CEPGA can be applied to all protein–ligand docking problem in Table 3. When comparing algorithm 1 with algorithm 2, the algorithm 1 is superior to the algorithm 2 if the p value is less than 0.05.

**Table 3:** Hypothesis test result

	CEPGA	LGA	GA	SODOCK	ABC
3ptb					
CEPGA	–	0.012	0.004	0.028	0.006
LGA	0.988	–	0.008	0.563	0.225
GA	0.996	0.992	–	0.995	0.688
SODOCK	0.972	0.437	0.005	–	0.100
ABC	0.994	0.775	0.312	0.900	–
1aha					
CEPGA	–	0.519	0.402	0.124	0.105
LGA	0.481	–	0.036	0.017	0.004
GA	0.598	0.964	–	0.342	0.260
SODOCK	0.976	0.983	0.658	–	0.470
ABC	0.895	0.996	0.740	0.530	–
3hvt					
CEPGA	–	0.035	0.007	0.023	0.013
LGA	0.965	–	0.205	0.324	0.150
GA	0.993	0.795	–	0.778	0.437
SODOCK	0.977	0.676	0.222	–	0.215
ABC	0.987	0.850	0.463	0.585	–
1phg					
CEPGA	–	0.036	0.008	0.041	0.017
LGA	0.964	–	0.016	0.624	0.450
GA	0.992	0.984	–	0.988	0.537
SODOCK	0.959	0.376	0.012	–	0.215
ABC	0.983	0.550	0.463	0.785	–
2mcp					
CEPGA	–	0.013	0.004	0.002	0.003
LGA	0.987	–	0.203	0.182	0.224
GA	0.996	0.797	–	0.492	0.610
SODOCK	0.997	0.818	0.508	–	0.640
ABC	0.995	0.776	0.390	0.360	–
1stp					
CEPGA	–	0.034	0.007	0.042	0.013
LGA	0.964	–	0.009	0.624	0.450
GA	0.993	0.991	–	0.992	0.487
SODOCK	0.958	0.376	0.008	–	0.215
ABC	0.987	0.550	0.513	0.785	–
6rnt					
CEPGA	–	0.035	0.008	0.029	0.010
LGA	0.965	–	0.018	0.368	0.127
GA	0.992	0.982	–	0.695	0.588
SODOCK	0.971	0.632	0.305	–	0.404
ABC	0.990	0.873	0.412	0.496	–
4dfr					
CEPGA	–	0.015	0.005	0.011	0.008
LGA	0.985	–	0.009	0.337	0.115
GA	0.995	0.991	–	0.986	0.685
SODOCK	0.989	0.663	0.014	–	0.142
ABC	0.992	0.885	0.315	0.858	–
1ets					
CEPGA	–	0.025	0.009	0.015	0.018
LGA	0.975	–	0.018	0.063	0.127
GA	0.991	0.982	–	0.595	0.688
SODOCK	0.985	0.937	0.405	–	0.504
ABC	0.982	0.873	0.312	0.496	–
1hri					
CEPGA	–	0.038	0.002	0.040	0.015
LGA	0.962	–	0.014	0.723	0.151
GA	0.998	0.986	–	0.982	0.637
SODOCK	0.960	0.277	0.012	–	0.020
ABC	0.985	0.849	0.363	0.980	–
1hvr					
CEPGA	–	0.043	0.005	0.011	0.009
LGA	0.957	–	0.038	0.177	0.044
GA	0.995	0.962	–	0.942	0.565
SODOCK	0.989	0.823	0.058	–	0.168
ABC	0.991	0.956	0.435	0.832	–
4hmg					
CEPGA	–	0.020	0.005	0.017	0.010
LGA	0.980	–	0.008	0.417	0.214
GA	0.995	0.992	–	0.988	0.900
SODOCK	0.983	0.583	0.012	–	0.240
ABC	0.990	0.786	0.100	0.760	–
1cdg					
CEPGA	–	0.017	0.006	0.044	0.005
LGA	0.983	–	0.117	0.763	0.105
GA	0.994	0.883	–	0.985	0.408
SODOCK	0.956	0.237	0.015	–	0.012
ABC	0.995	0.895	0.592	0.988	–
1htf					
CEPGA	–	0.148	0.023	0.640	0.015
LGA	0.852	–	0.027	0.883	0.151
GA	0.977	0.973	–	0.987	0.637
SODOCK	0.360	0.127	0.013	–	0.017
ABC	0.985	0.849	0.363	0.983	–
1glq					
CEPGA	–	0.045	0.009	0.049	0.042
LGA	0.955	–	0.018	0.163	0.227
GA	0.991	0.982	–	0.695	0.788
SODOCK	0.951	0.837	0.305	–	0.704
ABC	0.958	0.773	0.212	0.296	–
1tmn					
CEPGA	–	0.317	0.008	0.744	0.095
LGA	0.683	–	0.217	0.763	0.105
GA	0.992	0.783	–	0.905	0.408
SODOCK	0.256	0.237	0.005	–	0.012
ABC	0.995	0.895	0.592	0.988	–

## Discussion

Drug molecular design plays a decisive role in the development of drugs. Protein–ligand docking is the major method of computer aided drug design (Guedes et al. 2014; Huang and Zou 2010), which takes advantage of the combination of drug chemistry and computer technology to improve the efficiency of drug development (Zhao et al. 2008, 2011). The aim of protein–ligand docking is to find the best ligand conformation of a ligand against a protein target with the lowest energy (Bohlooli et al. 2017). many researchers have made great efforts to improve the power of the protein–ligand docking methods, such as simulated annealing (SA), genetic algorithm (GA) (Jones et al. 1997), Lamarckian genetic algorithm (LGA) (Fuhrmann et al. 2010), SODOCK (Chen et al. 2007), and artificial bee colony (ABC) (Uehara et al. 2015). However, the quality of the solutions that the existing algorithms obtain is insufficient. This paper illustrates a novel and robust optimization algorithm (CEPGA) for solving the protein–ligand docking problems with an aim to overcome the above-mentioned drawback.

An efficient docking method consists of two connected goals, which are the fitness accuracy (energy based) and the pose accuracy (root mean square deviation (RMSD) based) (Guo et al. 2014; Hu et al. 2004). For the fitness accuracy, the lower energy is associated with the greater binding activity which can also give rise to better drug efficiency. RMSD is utilized to determine whether two docked conformations are similar enough to be categorized into the same cluster. A docked conformation with a smaller RMSD is considered as a more accurate solution to the docking problem. Compared CEPGA with GA, LGA, SODOCK, and ABC (Castro-Alvarez et al. 2017; Feinstein and Brylinski 2015), Table 2 show that CEPGA has the best performance in the search for the lowest energy and the smallest RMSD of molecular docking conformations.

We also evaluate the performance of CEPGA in other aspects including convergence analysis, data distribution, and hypothesis test (Knowles et al. 2006) in comparison with GA, LGA, SODOCK, and ABC (Castro-Alvarez et al. 2017; Feinstein and Brylinski 2015). The convergence diagrams (Fig. 3) indicate that CEPGA is superior to other methods in terms of convergence rate and solution quality, and these figures also show that CEPGA can prevent premature convergence. For data distribution, as seen in box plots (Fig. 4), the medians of CEPGA are the lowest and its data are the most concentrated. This can demonstrate that CEPGA is a stable algorithm for protein–ligand docking. Hypothesis tests are showed in Table 3, and it can be obviously seen that CEPGA is better than other algorithms according the p value in the tables.

In conclusion, the paper presents the CEPGA which combines genetic algorithms, crossover elitist preservation (CEP), and local search method to extends the power of the GA_based algorithm for molecular docking problems. By using the CEP mechanism, the search algorithm not only can retain elitists to improve the efficiency of crossover, but also can get better energy value and RMSD. The five search methods, CEPGA, LGA, GA, SODOCK, and ABC are tested by experiments above. The results indicate that CEPGA has superior ability to the other four search algorithms in terms of robustness and efficiency. This suggests that CEPGA can enhance the applicability of AutoDock to docking problems.

## Abbreviations

CEP: crossover elitist preservation
CEPGA: genetic algorithm with crossover elitist preservation
SA: simulated annealing
GA: genetic algorithm
LGA: Lamarckian genetic algorithm
ABC: artificial bee colony algorithm
RMSD: root-mean-square deviation
